# An UltraMNIST classification benchmark to train CNNs for very large images

**DOI:** 10.1038/s41597-024-03587-4

**Published:** 2024-07-12

**Authors:** Deepak K. Gupta, Udbhav Bamba, Abhishek Thakur, Akash Gupta, Rohit Agarwal, Suraj Sharan, Ertugul Demir, Krishna Agarwal, Dilip K. Prasad

**Affiliations:** 1 Transmute AI Lab (Texmin Hub), Indian Institute of Technology, ISM Dhanbad, India; 2 Bio AI Lab, Department of Computer Science, UiT The Arctic University of Norway, Tromso, Norway; 3 Hugging Face Inc., Paris, 75002 France; 4 Global Maksimum Data & Information Technologies, Istanbul, Turkey; 5 Department of Physics and Technology, UiT The Arctic University of Norway, Tromso, Norway

**Keywords:** Computer science, Information technology

## Abstract

Current convolutional neural networks (CNNs) are not designed for large scientific images with rich multi-scale features, such as in satellite and microscopy domain. A new phase of development of CNNs especially designed for large images is awaited. However, application-independent high-quality and challenging datasets needed for such development are still missing. We present the ‘UltraMNIST dataset’ and associated benchmarks for this new research problem of ‘training CNNs for large images’. The dataset is simple, representative of wide-ranging challenges in scientific data, and easily customizable for different levels of complexity, smallest and largest features, and sizes of images. Two variants of the problem are discussed: standard version that facilitates the development of novel CNN methods for effective use of the best available GPU resources and the budget-aware version to promote the development of methods that work under constrained GPU memory. Several baselines are presented and the effect of reduced resolution is studied. The presented benchmark dataset and baselines will hopefully trigger the development of new CNN methods for large scientific images.

## Background & Summary

Convolutional neural networks (CNN) are arguably one of the biggest breakthroughs for image processing, especially due to their capability of extracting information beyond what can be achieved by conventional computer vision methods (see comprehensive reviews in^1–3^). They have been designed for images of the everyday world acquired using consumer cameras, and have been performance leaders for quite sometime. They have also started creating impact in scientific domain with applications in medical imaging^4,5^, geophysical tomography and imaging^6–9^, design optimization of structures^10,11^, microscopy^12–15^, among others. However, there are certain limitations of CNN that still need attention when applied in scientific domains, and we address one fundamentally limiting aspect in this paper.

There is an evolution in scientific domains such as microscopy^16,17^, earth sciences^18,19^, and space observations using the recently launched James Webb Space Telescope, where huge images are created due to the latest scientific technologies. Here, we will use super-resolution microscopy (more popularly referred to as nanoscopy) as a case of discussion, which won the Nobel Prize in Chemistry in 2014. Indeed, deep learning is considered of interest in these domains as well, in particular in the microscopy domain^12–15^. However, the big data challenge of applying CNNs to analyze nanoscopy images is immense, as we demonstrate here.

**Fig. 1:**
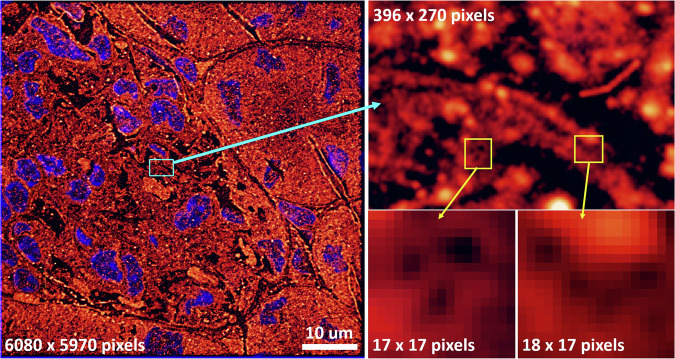
On the left is a nanoscopy image of a mouse kidney cryo-section approximately 1/12th of the area of a single field-of-view of the microscope, chosen to illustrate the level of details at different scales. The bottom right images show that the smallest features in the image of relevance to biological factors can be as small as a few pixels (here 5-8 pixels for the holes)^20^.

High content nanoscopy involves taking nanoscopy images of several adjacent fields-of-view and stitching them side-by-side to have a full perspective of the biological sample, such as a patient’s tissue biopsy, put under the microscope. There is information at multiple scales embedded in these microscopy images^20^ (see Fig. 1), with the smallest scale of features being only a few pixels in size. Indeed, such dimensions of images and levels of details are a challenge for CNNs, which are often conventionally designed for a fixed input size, such as 128 × 128 pixels or up to 512 × 512 pixels.

With these large images, there are limited ways to approach the problem using current CNN methods. It is most common to reduce the resolution of the image, however, the small scale features are lost, and this can completely change the associated semantic context. Another popular strategy is to break the image into a set of partially overlapping tiles and train CNN with subsets of these tiles. The limitation is that the semantic link between the tiles is not known a priori, and disconnecting them leads to a loss of semantic information across the image. This issue can be resolved to a certain extent using a two-stage training pipeline where the original image is first projected onto a low-dimensional latent space, and then this compressed version is used for training. However, this also involves a significant information loss and can only work for up to a certain level of the increased size of the input. With the extreme variation in the scale of the features, choosing the right tile size is also hard. Multiple CNNs could be used to handle different scales, or methods need to be developed to collate information from the outputs of all these CNNs into a single coherent output. The challenges in 3D and 3D with temporal data are further compounding in terms of the handling of data, designing the right architectures, and incorporating spatial and temporal correlations of value.

**Fig. 2:**
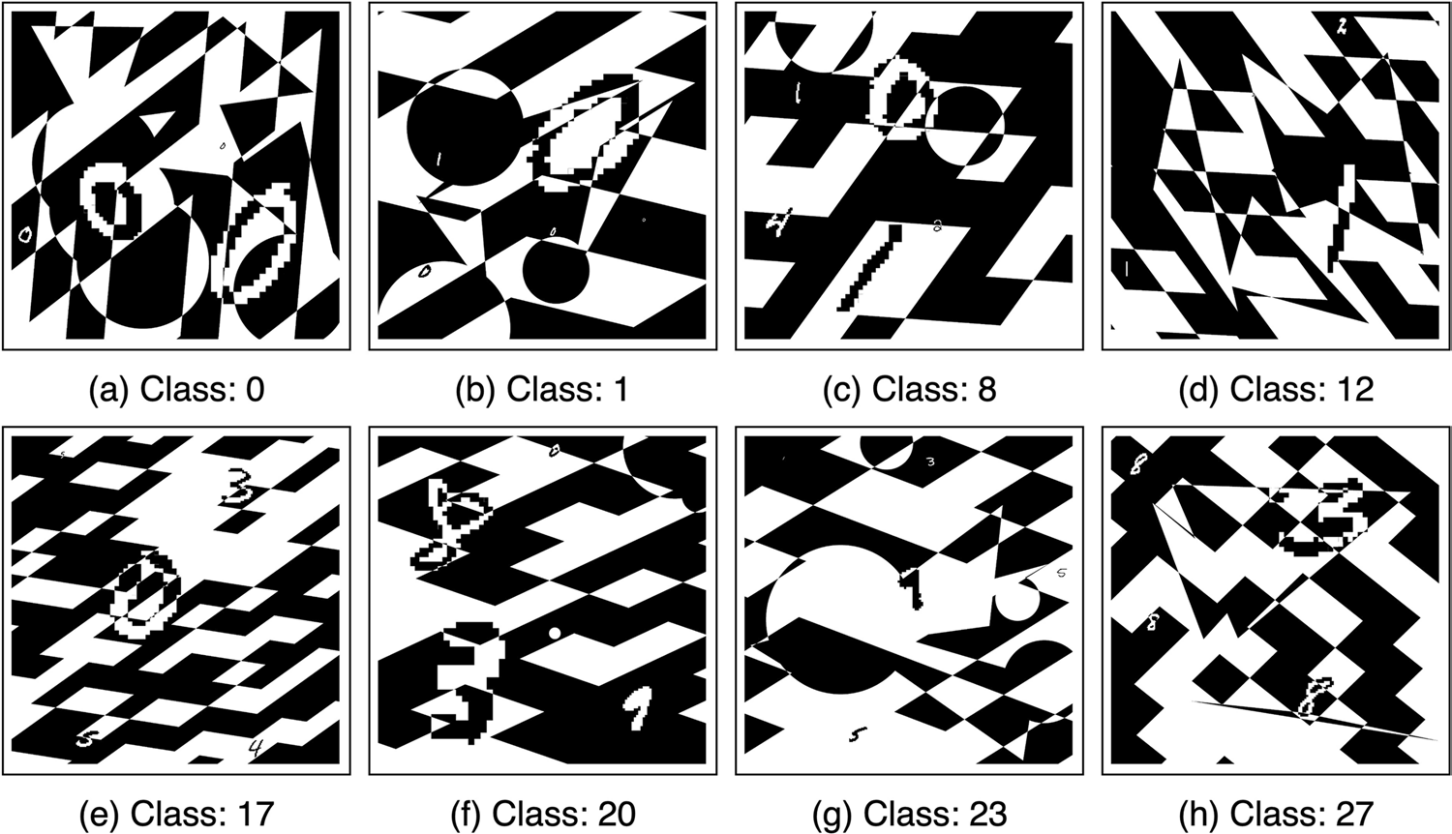
Example images for 8 out of the 28 classes of UltraMNIST dataset^21^.

To promote the development of solutions to the problem outlined above, we introduce the ‘UltraMNIST dataset’^21^, a simple yet good representative dataset to develop CNN training methods for handling very large images. UltraMNIST data samples are constructed using the popular MNIST digits^22^ with some additional modalities. Each sample image is a 4000 × 4000 grid and contains 3-5 MNIST digits varying in size from as small as 14 × 14 pixels to as large as 2000 × 2000 pixels. Example samples from this dataset are shown in Fig. 2.

Further, a complex stochastically sampled background is added to restrict explicit sampling of the MNIST digits from the full-scale UltraMNIST sample. Details related to the synthesis of the background are described in a later section of this paper. The machine learning problem to be solved here is to predict the sum of all the digits that exist in every image. This poses the problem of building a semantic relation between distant parts of the images such that the features in different parts are significantly different in terms of scale.

We present two variations of the UltraMNIST benchmark. First is the standard version, where there is no restriction on the GPU or TPU memory to be employed for the problem. This is intended to promote the efficient use of the latest hardware and identify the best CNN training strategies that could even involve splitting the training of a large image on a distributed set of GPUs. Alternatively, it could also be possible to develop smart training, or downsampling strategies that deliver good performance with even simple models such as ResNet-50, and the standard UltraMNIST classification benchmark provides this opportunity. On a different note, UltraMNIST digits are representative of even larger images, which could be as large as 50000 × 50000 pixels and more, and even the state-of-the-art GPU resources cannot work with this resolution. To mimic this challenge of the real problems, we introduce a ‘budget-aware variant’ of the UltraMNIST classification problem. This benchmark puts a constraint on the GPU memory that can be used by the training and inference steps of any novel solution to the UltraMNIST classification problem. In this paper, we choose a memory budget of 11 GB to build the first baselines.

To accelerate the development of useful models, we present several baseline solutions for the two variants. These methods explore how well the current state-of-the-art classification models work for the presented problem. To keep the baselines competitive, we hosted a Kaggle community competition at https://www.kaggle.com/c/ultra-mnist, where we invited the community of data scientists to propose novel solutions to UltraMNIST classification. Most of the solutions are based along the lines of reducing the resolution of the large images and seeing the extent of drop observed with different SOTA models. To understand the efficacy of the machine learning models, we also present human baseline scores computed based on the labeling of subsets of the test data by humans. Further, solutions are presented for different resolutions of the input for popular models such as ResNets and EfficientNets.

In the literature, several datasets are associated with the MNIST datasets, including Fashion-MNIST^23^, MedMNIST^24^, and EMNIST^25^. This paragraph focuses on these datasets and examines their deviations from UltraMNIST. Fashion-MNIST comprises a dataset of 70,000 fashion products distributed across 10 categories, with each category containing 7,000 images. Each image is a 28 × 28 grayscale image. Fashion-MNIST mirrors the MNIST dataset in terms of image size, data format, and the division of training and test splits. This structural similarity allows for the direct substitution of the MNIST dataset with Fashion-MNIST by just altering the dataset’s URL, thereby simplifying the benchmarking process. The MedMNIST dataset comprises 10 pre-processed, open datasets from the medical domain, each containing images ranging from 100 to 100,000. Each image has a resolution of 28 × 28, consistent with the MNIST dataset. The recent development of MedMNIST v2^26^ has expanded these offerings by incorporating 8 additional bioimage datasets. Similar to the original 10 2-dimensional datasets in MedMNIST, version 2 introduces 2 new 2-dimensional datasets and 6 3-dimensional datasets. EMNIST represents an expanded version of the MNIST dataset, incorporating letters in addition to digits. These letters are sourced from the original MNIST dataset’s foundational material, known as the NIST Special Database 19. The EMNIST dataset retains the same image structure and parameters as its predecessor, the MNIST dataset, thereby ensuring compatibility with existing MNIST classifiers. The above datasets are related to MNIST due to their similarity in image size and compatibility with existing MNIST classifiers. However, UltraMNIST utilizes the digits from the MNIST dataset to generate images of 4000 × 4000 pixels in order to advance the development of CNNs for processing large images.

### Contributions

- We present the ‘UltraMNIST dataset’^21^, a simple yet good representative benchmark dataset for the problem outlined above. UltraMNIST has been designed using the popular MNIST digits with additional levels of complexity added to capture the real-world challenges.
- In this paper, we introduce a novel research problem of training CNN models for very large images. Referred to as ‘UltraMNIST classification’ (See latest updates at https://transmuteai.github.io/ultramnist/), this problem is designed to innovate in terms of novel CNN training pipelines that can facilitate the efficient handling of very large images under limited GPU memory constraints.
- We present two variants of the classification problem: ‘UltraMNIST classification’ and ‘Budget-aware UltraMNIST classification’. The standard UltraMNIST classification benchmark is intended to facilitate the development of novel CNN training methods that make effective use of the best available GPU resources. The budget-aware variant is intended to promote the development of methods that work under constrained GPU memory.
- For the development of competitive solutions to the UltraMNIST classification problem, we present several baseline models for the standard benchmark and its budget-aware variant.
- We study the effect of reducing the resolution on the performance of the model, and results are presented for baseline models involving pretrained backbones from among the popular state-of-the-art models.

## UltraMNIST dataset

### Data description

UltraMNIST^21^ comprises a total of 28000 images of size 4000 × 4000 pixels in the training set as well as 28000 images in the test set. Each UltraMNIST sample comprises 3 to 5 MNIST digits, and the sum of the digits forms the label of the sample. The digits are sampled such that the minimum sum is 0 and the maximum sum is 27. Beyond the MNIST digits, UltraMNIST samples also comprise random shapes and a randomly oriented checkerboard pattern for additional complexity. The goal is to build a classifier model that classifies each UltraMNIST sample into one of the 28 classes based on the sum of the digits contained in it.

#### Why 4000 × 4000 pixels?

This choice comes from identifying a balance between the complexity of the problem that this paper intends to pose and the ease of conducting preliminary research experiments toward solving it. It is straightforward to scale-up the image size to even 100,000 × 100,000 pixels with minimal effort, and it would have represented the posed problem even better. However, even loading such images into the memory is challenging with conventional computational resources, let alone building deep learning solutions that can handle it. On the other side, we could also choose images of size smaller than the current one, however, the best solution reported in the paper as well as some in-house experiments revealed that smaller image sizes could be decently handled by the current CNN training strategies using the commonly available compute resources.

#### Why MNIST digits?

The primary reason to use MNIST as the base dataset is that the digits of this dataset are relatively simple to classify and require only a few discriminative features to be represented well. With the additional complexities, the MNIST digits clearly suffice the requirement of complexity needed for our task. With this, we reduce the load of building a too complex and heavy discriminative model to identify the objects in our UltraMNIST samples so that more effort can be put into how the CNN training pipeline itself can be improved for handling our large images.

#### Why 3-5 digits per UltraMNIST image?

For semantically connecting different parts of the image, we needed at least two samples. To add to the difficulty level, we wanted to choose a higher number, however, going beyond 5 added too much clutter, hence a range of 3 to 5 was chosen.

#### Why only the MNIST training set?

For the synthesis of UltraMNIST digits for the training as well as the test sets, we used only the MNIST training set. This was done to reduce the load on generalising towards the identification of the MNIST digits. The goal for UltraMNIST benchmarking is to handle the semantic correspondence across the large image space, to be robust to large-scale variations, as well as be able to clearly identify the relevant information in the complex background. To ensure that any dip in a model’s performance is only because of its limitation in handling one of these factors, we chose to sample the MNIST digits from only the training set. For each UltraMNIST sample, the background contains a random checkerboard where the size of boxes is variable across samples, rotation and shear transformation by random amounts, as well as an additional random number of perturbations in the form of a selected set of shapes. On top of this, the colour of the digits is locally adjusted according to the background, leading to digits that are not constant in colour throughout. Moreover, the combination of MNIST digits is randomly chosen. Based on the above mentioned factors, we believe that the samples are already diverse across the training and test sets. This was also reflected in the large gap between the models’ performance on train and test sets for the experiments conducted.

#### Why extreme scale contrasts?

The motivation for using extreme scale contrasts of as much as 1:140 comes from the requirements posed by real-world problems. Additionally, the scale contrast prohibits reducing the resolution of the images to a very low order since the small digits would disappear then, and the semantic context would be lost. The too large digits are chosen such that the receptive field of the kernels employed in current CNN training schemes cannot capture them fully.

#### Why background clutter?

If the digits are left on an empty white background, they can be individually extracted using simple pre-processing methods and brought to the same scale. While this makes the problem easy to solve, it will completely ruin the whole motivation of this research problem. To circumvent this issue, we have added a complex background that cannot be simulated easily and ensure that any preprocessing object detection methods are not used.

### UltraMNIST benchmark

UltraMNIST benchmark is a classification problem that aims at assigning each UltraMNIST digit to one of the 28 classes defined earlier. The foundation of the UltraMNIST classification benchmark is built around the popular MNIST classification problem with added levels of complexity to pose the challenges of very large images carrying distributed semantic context. The goal is to develop novel CNN training strategies to achieve the highest classification accuracy score on the test subset of UltraMNIST. This benchmark promotes two different directions of research: First is to modify the current CNN training pipelines such that the current classification models can be used for the classification of very large images. The second is to modify the architecture of current CNN models to adapt their receptive fields for very large images and deploy them on the most recent state-of-the-art GPU hardware. Scientific developments along both these lines answer the question posed by this benchmark and are to be treated as acceptable solutions. Based on the description above, we formally describe this benchmark problem as follows.

*UltraMNIST Classification Benchmark. Find a solution to the classification of UltraMNIST digits into one of the target classes such that the accuracy score obtained over the test dataset is maximized*.

Note that the use of the MNIST dataset in any form during training or testing of UltraMNIST digits is prohibited. The primary reason is that the UltraMNIST digits are designed to mimic real-world problems where a single object in a large image might not necessarily carry any semantic context, but collectively multiple objects in that image make a meaning. We pose a similar problem where no a priori mathematical information on the MNIST digits is to be hard baked into the model. The trained models should not be explicitly made to understand the MNIST digits, rather only the labels of the UltraMNIST digits should be used to guide the supervised learning process. For example, a single digit ‘5’ in our images should not be interpreted as ‘5’ since there is no information as such in the data. However, occurrences of sets such as ‘5, 0, 0’, ‘4, 1, 0’ and ‘3, 1, 1’ are to be labeled as ‘5’.

On a different note, we also accepted solutions to the unconstrained version of this problem in the challenge hosted on the Kaggle platform, and several interesting solutions were proposed that used the original MNIST digits. This is briefly discussed in the Technical Validation section of this paper.

### Budget-aware UltraMNIST Benchmark

Budget-aware UltraMNIST refers to a similar classification problem as described above but with an additional constraint on the usage of GPU memory during the training and inference of the deep learning model. With the additional constraint, we hope that novel solutions will be proposed to develop CNN training pipelines specific to available hardware specifications. In general, hardware-related developments are slower than software ones, and it is not an easy task to upgrade the GPU hardware based on the requirements posed by changes in the CNN models. We propose to investigate methods that alleviate this challenge by transforming the load on computational memory into increased computational time. This would hopefully allow us to train CNN models for very large images with even constrained GPU resources. An added advantage would be that the novel strategies developed using this benchmark could help to even scale the current large CNN models for low memory devices. In this paper, we present the case of the GPU memory budget of 11 GB. However, the concept of budget-aware classification of UltraMNIST holds for any prescribed budget, and we additionally present scores for a GPU memory budget of 24 GB. Furthermore, we express this problem more formally as follows.

*Budget-aware UltraMNIST Classification Benchmark. With the maximum GPU memory usage of N GB during the training and inference stages, find a solution to the classification of UltraMNIST digits into one of the target classes such that the accuracy score obtained over the test dataset is maximized*.

## Methods

### Data creation

**Fig. 3:**
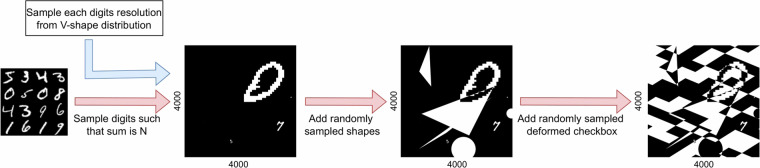
Schematic workflow for the generation of UltraMNIST^21^ samples.

The process of creation of the UltraMNIST samples is schematically described in Fig. 3. First, 3 to 5 digits are randomly sampled from the MNIST training set. The reason to use only the MNIST training set for generating UltraMNIST train, as well as test samples, is described in the Discussion section of this paper. Next, for each MNIST, a scale value is sampled from a V-shape distribution, where the two extremes are 14 × 14 and 2128 × 2128. For upscaling above 28 × 28, the scale value is used, whereas for downscaling to 14 × 14, the nearest neighbor interpolation is employed. Using these scales, the sampled 28 × 28 MNIST image (/digits) is placed randomly on a canvas of 4000 × 4000 resolution image. Before placing the digits, it is checked for any overlap. If it overlaps, then the digit is not added to the canvas. This results in an image without any overlap.

To further increase the difficulty of UltraMNIST and to make it ideal for real-world benchmarking, we add a complex background in each image. The addition of background is a two-fold process where, firstly, we sample random circles and triangles and overlay them randomly using the XOR operation on the canvas. We sample each shape randomly from zero to five. Further, an augmented checkerboard pattern is sampled, which is then overlaid on the canvas. Augmentations on the checkerboard include rotations by ± 45% and shear of ± 50%.

### Implementation details

#### Data split

For all the experiments (unless stated otherwise), we use a split of 90% and 10% for the train and validation sets, respectively. This validation set is used to perform early stopping of the training process and as well as select a model to test on the test set.

#### Data resizing

For the baseline scores reported in the paper, downsampled versions of UltraMNIST images are used. However, we have observed that if the 4000 × 4000 are directly downsampled to 512 × 512 or lower in one step, there is a detrimental effect on the performance of the model. To circumvent this issue, we conduct progressive downsampling for the case of small images. If the image size is greater than or equal to 512 × 512, we downsample in one step, however, for lower resolutions, we first obtain the image resolution of 512 × 512, and then downsample it further to achieve the desired lower resolution.

#### Training details

All our baseline networks are trained using Adam optimizer^27^ and exponential learning rate scheduler. Further, we don’t apply any data pre-processing and augmentations apart from resizing and normalization. For the case of budget-constrained UltraMNIST experiments, the batch-size of networks are scaled such that they would fill the maximum GPU memory. Further, we did random hyper-parameter search to get the best set of learning rate and gamma values for the optimizer and the scheduler, respectively. The best sets of hyper-parameters for 11 GB and 24 GB are reported in Table 1 and Table 2, respectively. For the ENB0-2048 and ENB0-4000, the gamma value was set to 1 and batch-size was fixed to 4 and 1, the learning rate of 1.4 × 10^−4^ and 8 × 10^−4^ were used, respectively.

**Table 1:** Hyper-parameters used for 11 GB benchmark.

Model	Image Size	Learning Rate	Gamma	Batch-size
EfficientNetB0	256	1 × 10^−3^	0.8	76
	512	1 × 10^−3^	0.8	20
	1024	1 × 10^−4^	0.94	4
EfficientNetB3	256	1 × 10^−3^	0.8	40
	512	5 × 10^−4^	0.97	12
	1024	5 × 10^−5^	0.97	2
ResNet-50	256	2 × 10^−3^	1	68
	512	1 × 10^−3^	0.94	18
	1024	1 × 10^−3^	0.97	3

**Table 2:** Hyper-parameters used for 24 GB benchmark.

Model	Image Size	Learning Rate	Gamma	Batch-size
EfficientNetB0	256	4 × 10^−3^	0.98	170
	512	2 × 10^−3^	0.98	46
	1024	2 × 10^−3^	0.98	10
EfficientNetB3	256	2 × 10^−3^	0.98	92
	512	8 × 10^−4^	0.98	32
	1024	2 × 10^−3^	0.98	6
ResNet-50	256	2 × 10^−3^	1	140
	512	2 × 10^−3^	1	48
	1024	3 × 10^−4^	0.98	10

#### Hardware configuration

All experiments (except MBNet-demir, EffNetTPU-vexcoz, and those with 2048 and 4000 resolution) were performed on 2080Ti (11GB) and 3090Ti (24GB) GPUs. Both the machines had 16 GB RAM and 4 core processors. EffNetTPU-vexcoz method used a Kaggle publicly available TPUv3 instance, whereas MBNet-demir used a Kaggle publicly available P100 GPU (16 GB) instance. Both these instances have 12 GB RAM and 2 core processors. Experiments with resolutions 2048 and 4000 were performed on V100 (32 GB) GPU with 256 GB RAM and 64 Core CPU.

## Data Records

The UltraMNIST dataset is accessible at 10.18710/4F4KJS^21^, and the code used to generate it can be accessed at https://github.com/transmuteAI/ultramnist.

Here, we present the usage notes for readers accessing and using the data records. There are 56000 image files in JPEG format, which are split into a train folder and a test folder, each containing 28000 files. Further usage guidance is also available at 10.18710/4F4KJS in the readme file named 00_ReadMe.txt.

**Fig. 4:**
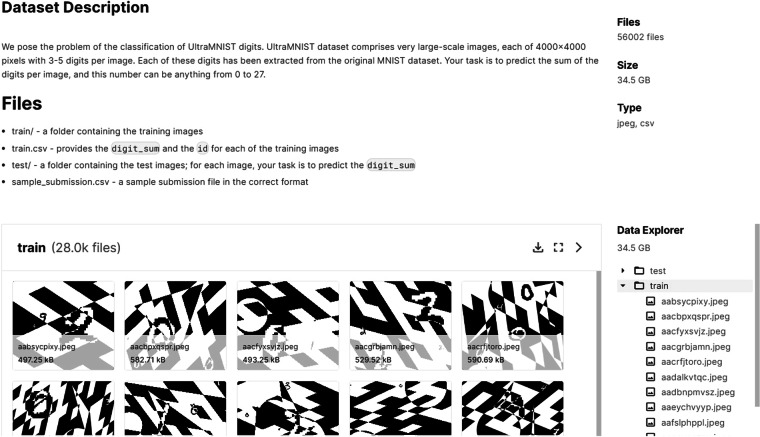
Screenshot of the Kaggle page of UltraMNIST^21^ from where the data can be downloaded. The data is available in the right pane under the heading **Data Explorer**. Clicking on the ’train’ folder results into a view of the images in the center panel as well as the list of image names under the folder.

These folders are also available in the right pane of https://www.kaggle.com/competitions/ultra-mnist/dataunder the heading **Data Explorer** in an easy-to-view format. The images in the folder can be easily viewed by clicking on the folder in the viewing pane in the center of the screen. These aspects are shown in the screenshot in Fig. 4. Individual images can be downloaded by selecting the image in the viewing pane or the right panel.

Alternatively, the entire dataset can be downloaded at once from the Kaggle URL or the DOI link. However, we alert the users about the large size of the dataset (34.5 GB). The Kaggle cloud ensures the continuity of the download despite the large size of the dataset.

There is one more file in the format. csv outside the folders, which is of interest to the users. It is named ‘train.csv’. It contains two columns, one with the image file names in the training dataset and the other with the sum of digits in the corresponding images. The second column is, therefore, the expected output of the classification model.

Now we describe the codeset in https://github.com/transmuteAI/ultramnist. Python 3.9.12 will be needed. We recommend setting up a virtual environment with pip. Further, the users must install the packages listed in the file named “requirements.txt”. Once the packages are installed, the codeset is ready for execution. The code set contains the codes for generating new data, training for the classification problem, and testing the model. All configurations concerning data, model, training, etc., can be called using the command line arguments. To create a dataset of images of any specified size, the file “data_generator.py” has to be used. The syntax is provided on the GitHub page.

## Technical Validation

### Human Baseline

UltraMNIST poses the challenge of identifying very small MNIST digits in the midst of complex background noise. Unlike the original MNIST digits, our UltraMNIST is also hard for humans to label. To validate this, we first present our human baseline developed for this dataset. For this, we approached several people who were not involved in the curation process. We developed our human baselines for two different variants: guided and unguided. For the unguided variant, we handed over the UltraMNIST images to the participants and asked them to assign a label between 0-27 based on the sum of digits that they observed in the image. It was ensured that no additional information was provided for this variant of the baseline. For the guided variant, we additionally informed the participants about the contrasting scale of the digits from being extremely small to very large. Further, each participant from this pool was shown the entire set of MNIST digits that were used for curation and informed to only search for 3-5 of these digits in each image and sum them up to form the corresponding label. Each participant was asked to label between 20-40 random images from the test set, and the pool of candidates for the two variants was completely different. In total, we got a subset of 1000 images labeled for each pool, and accuracy scores of 49.3% and 52.1% were obtained for the unguided and guided variants, respectively (see Table 3).

**Table 3:** Performance scores for the various baselines on UltraMNIST^21^ and Budget-aware UltraMNIST benchmarks.

Method	Backbone	Input size	Acc. (%)	Acc.@11 GB (%)
Random baseline	—	—	3.6	—
Human baseline (unguided)	—	—	49.3	—
Human baseline (guided)	—	—	52.1	—
RN-256	ResNet-50	256 × 256	8.9	8.9
RN-512	ResNet-50	512 × 512	24.5	24.5
RN-1024	ResNet-50	1024 × 1024	31.3	31.3
ENB0-256	EfficientNet-B0	256 × 256	20.4	18.6
ENB0-512	EfficientNet-B0	512 × 512	49.8	43.9
ENB0-1024	EfficientNet-B0	1024 × 1024	49.0	49.0
ENB0-2048	EfficientNet-B0	2048 × 2048	75.1	—
ENB0-4000	EfficientNet-B0	4000 × 4000	8.2	—
ENB3-256	EfficientNet-B3	256 × 256	30.3	26.7
ENB3-512	EfficientNet-B3	512 × 512	72.3	**69.1**
ENB3-1024	EfficientNet-B3	1024 × 1024	79.1	54.5
MBNet-demir	MobileNet-V3	1280 × 1280	86.2	—
EffNetTPU-vecxoz	EfficientNet-B7	1536 × 1536	**93.8**	—

For the memory budget, we choose an upper limit of 11 GB GPU memory for the model training and inference tasks.

### Deep CNN models

We also present here a set of baselines built using some state-of-the-art models from the domain of image classification. To keep these baselines competitive, we hosted a community competition on the Kaggle platform where data scientists from across the globe could participate and submit solutions to the UltraMNIST classification problem. Among the solutions that satisfied the criteria outlined for the UltraMNIST benchmark, we either directly used them or added some fine-tuning to improve them further. Further, we also adapted some of the solutions to satisfy the budget-constraint described in the Budget-aware UltraMNIST benchmark. All scores related to the benchmarks are reported in Table 3. We describe the details related to each of these methods below.

#### ResNet and EfficientNet variants at several resolutions

As first CNN baselines, we present models using the popular image classification backbones, namely ResNet-50^28^, EfficientNet-B0^29^ and EfficientNet-B3. Three variants of image resolution are mainly tested: 256 × 256, 512 × 512, and 1024 × 1024. With EfficientNetB0, we also test resolutions of 2048 × 2048 and 4000 × 4000 to investigate the behaviour of current CNN models when using high-resolution inputs. We use the method naming convention in the format ‘BACKBONE-IMAGE WIDTH’, thereby RN-256 denotes an image classifier trained on images of resolution 256 × 256. See Table 3 for the full set of methods. Datasets at different resolutions have been created by resizing the original 4000 × 4000 images using inter-area interpolation. To avoid any excessive loss of information, images are progressively downscaled to achieve the desired resolution. For all the methods, the output from the backbone is mapped to 28 predefined classes using two fully-connected layers with a ReLU activation mapping between them.

#### MBNet-demir

This method is inspired from the approach presented in^30^, and uses an implicit resizing network instead of the conventional interpolation algorithms such as bilinear and bicubic. Further, this classification method uses a MobileNet-V3 backbone, and the resizing network is used to produce a more ‘CNN friendly’ representation of the image, which is in turn fed to the backbone as input. The advantage is that the information related to smaller objects is preserved, which the traditional approaches generally fail at. The resizing network, consisting of bilinear resizers, directly skips the network and adds the resized image to the output of the other branch. The other branch uses 2D convolutions as a bottleneck and a user-defined number of typical residual blocks. The skip connections of the network provide an easier learning process.

#### EffNetTPU-vexcoz

This method involves building a classification model using the EfficientNet-B7 backbone and fine-tuning it on the UltraMNIST training set. It involves two stages of training. During the first stage, the model is trained with images of resolution 1024 × 1024, and in the second stage, training is performed at an image resolution of 1536 × 1536. For the two stages, learning rates are set to 5 × 10^−4^ and 10^−4^ with a reduction on the plateau. For the purpose of augmentation, image inversion is used and new images are created by flipping pixel values on the grayscale axis.

#### Budget-constraint of 24 GB GPU memory

We report here additional results for the case where a constraint of 24 GB is set on the maximum GPU memory to be used during the training and inference processes by the model. Results related to this experiment are reported in Table 4. Note that the use of higher GPU memory in our case implies a larger batch size, and this may not necessarily increase the performance of the model. Thus, we report two different scores for the 24 GB GPU memory budget. First is Acc^*^@24, where the full GPU memory is utilized through scaling the batch size. Another is Acc@24, which denotes the best performance score with a GPU memory of 24 GB or less.

**Table 4:** Performance scores for various baselines on UltraMNIST^21^ benchmark. Here Acc^*^@24 GB % denotes the accuracy score in % obtained for full utilization of 24 GB GPU memory. Further, Acc@24 GB % denotes the best accuracy score for the GPU utilization of 24 GB memory or even less (e.g., 16 GB, 11 GB, etc).

Method	Backbone	Input size	Acc.^*^@24 GB (%)	Acc.@24 GB (%)
RN-256	ResNet-50	256 × 256	5.2	8.9
RN-512	ResNet-50	512 × 512	24.8	24.8
ENB0-256	EfficientNet-B0	256 × 256	20.4	20.4
ENB0-512	EfficientNet-B0	512 × 512	49.9	49.9
ENB0-1024	EfficientNet-B0	1024 × 1024	52.3	52.3
ENB3-256	EfficientNet-B3	256 × 256	30.3	30.3
ENB3-512	EfficientNet-B3	512 × 512	67.5	69.1
MBNet-demir	MobileNet-V3	1280 × 1280	—	**86.2**

Looking at the results, we see cases where utilizing the full GPU memory does not lead to increased performance. On the contrary, we see that for RN-256 and ENB3-512, performance drops. For RN-256, we have observed that the discriminative power of the model is not good enough to capture the fine details of the images. Due to extreme downscaling to 256 × 256, the small digits are hard to identify, and this leads to lower performance of RN-256. For the case of larger batch size, we believe that with further tuning of the learning rate and its decay, the performance gap between Acc^*^@24 GB and Acc@24 GB could be eliminated. In terms of the overall best for a 24 GB budget, we have MBNet-demir, which uses MobileNet-V3 and only utilizes a maximum memory of 16 GB.

#### Analysis of failure cases

To get a better understanding of when and why the model tends to mostly fail, we analyzed some of the failure cases for the results obtained on the validation set using ENB0-256 and ENB0-512 models. For this purpose, we randomly sampled 20 cases for each model where the prediction of the model was incorrect. In both cases, we observed that the model is able to identify the larger digits even in the midst of huge clutter. However, most of the failure cases happen in the presence of the small digits. In the case of 256 × 256, the occurrence of small digits makes the recognition extremely hard, due to which we see a significant drop in performance for ENB0-256. This clearly indicates that downsampling is not the best way to obtain maximum performance, and we need to identify ways to train CNN methods with images that undergo no downsampling.

#### Effect of random seed

the UltraMNIST dataset contains significant noise in each image. Moreover, the occurrence of extremely small digits together with the larger ones makes the performance of the model very sensitive to the training process. In our experience, even small differences in the training regime could significantly affect performance. To quantify this effect, we conducted a small sensitivity analysis test to investigate how much the accuracy of the model is affected by the random seed. For this, we used the EfficientNetB0 architecture with image resolutions of 256 × 256 and 512 × 512. We found that ENB0-256 obtained an accuracy of 18.45 ± 1.6% whereas ENB0-512 got 43.85 ± 4.7% over 3 runs. Clearly, there is a huge variance in the performance of the models for the variation in the random seed, and this has to do with the complexity of the information in the UltraMNIST samples. The high standard deviation in these experiments shows that we need better high-resolution image training pipelines, and the conventional pipelines are not well suited for such tasks, at least from the stability perspective. We intend to conduct more detailed research on this aspect as a part of our future work.

#### Unconstrained UltraMNIST classification

The unconstrained variant of our UltraMNIST competition refers to the pool where no restrictions are imposed. This implies that the candidates are free to use the MNIST dataset for model training, transform UltraMNIST into an object detection problem to identify each digit separately, and then build a model that learns to compute the summation. The use of MNIST digits allows the construction of a replica of UltraMNIST, where the bounding box around each digit is known for the training set, and object detectors can be trained. With extreme engineering, participants were able to score accuracy scores of even as high as 99%.

#### Additional visualizations

**Fig. 5:**
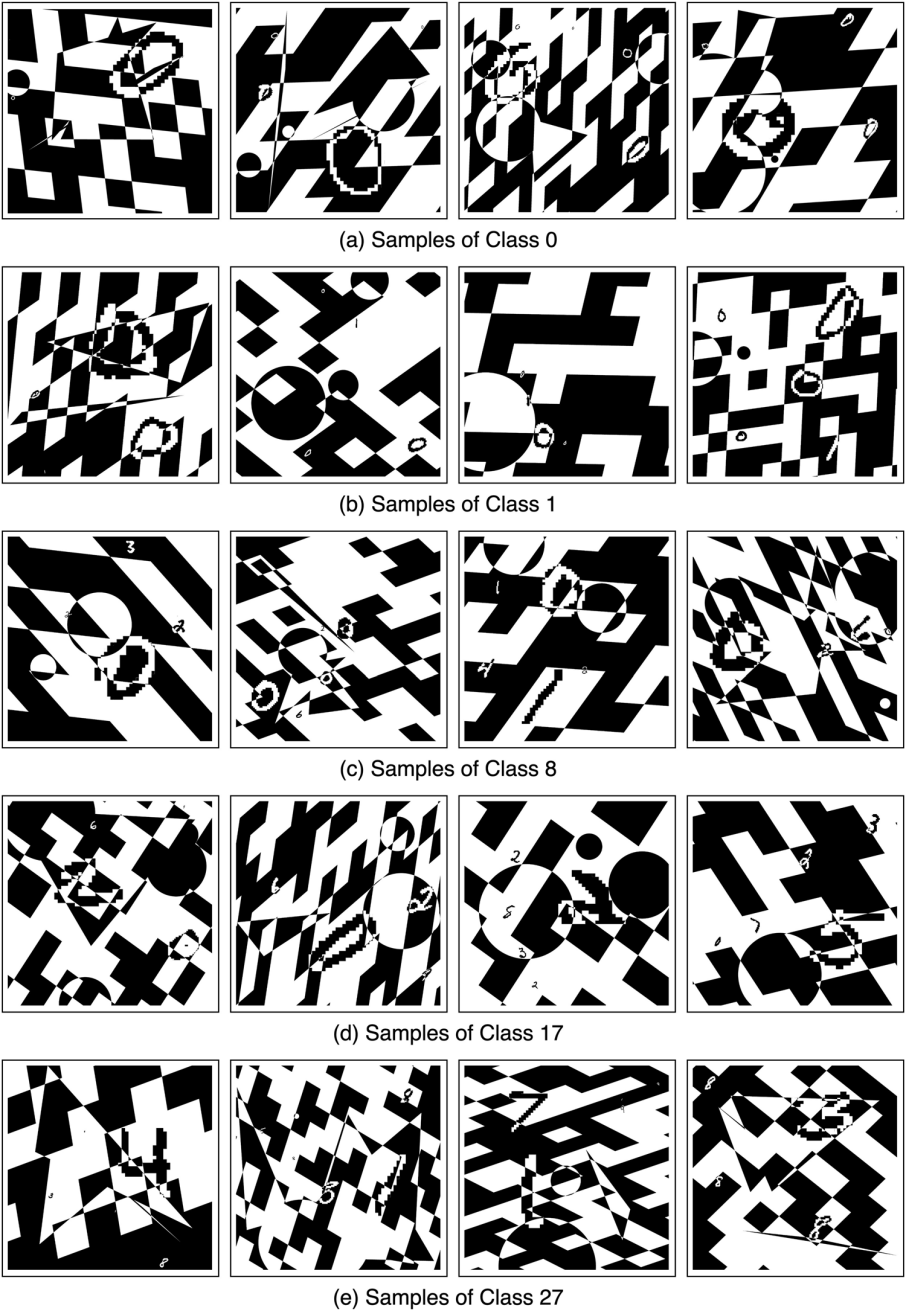
Examples from various classes of UltraMNNIST dataset^21^.

For the visualization of additional samples from different classes of the UltraMNIST dataset, please refer to Fig. 5.

#### Summary of the technical validation

As anticipated, reducing the image resolution too much has a large adverse effect on its performance. This is clear from the results reported for the image size of 256 × 256 pixels for the various methods. Even with the use of the EfficientNet-B3 backbone, the maximum accuracy score achieved is only 30%. For ResNet-50, it goes to less than 10%, clearly implying a significant loss of information at this reduced resolution. For ENB0-2048, the best accuracy obtained is 75.1%. Compared to the score for 512 × 512, we see a significant boost in the performance scores, clearly indicating that avoiding too much downsampling better facilitates the identification of the digits and leads to improved performance. However, as depicted in Table 3, these require more powerful computational resources. In a constrained GPU budget setting, only a very small batch size is possible for the training sets of such cases, and this leads to a dip in performance. This can be seen from the scores of ENB3-512 and ENB3-1024, where ENB3-512 outperforms the latter.

We further see that the EffNetTPU-vecxoz method significantly outperforms the other baselines. The primary reason for this success is the utilization of more than one image resolution in the training pipeline. Moreover, this method uses very high-resolution images in the second stage and utilizes TPU resources to fine-tune EfficientNet-B7. Nevertheless, such scalability of resources can happen only to a limited extent, and this clearly is not the final solution that we aim for the UltraMNIST benchmark, especially its budget-aware variant. On the positive side, the take away from this baseline is that in case we choose to work with reduced image resolutions, a direction would be to use more than one image resolution and build a unified pipeline. As a future research direction, this could also be something to investigate further.

While we have analyzed the performance of several baseline methods and their combinations with different input sizes, it is also of interest to study the challenges posed by the semantic content of the UltraMNIST samples. We look at the results of ENB3-256 and ENB3-512 and primarily analyze the failure cases. The obvious observation is that samples with smaller digits are relatively harder for both models to handle. Interestingly, the formulation of the UltraMNIST classification problem strictly enforces that all digits are recognized correctly, and the occurrence of even one small digit makes the classification hard. At this resolution, the largest digits are still within the scope of the receptive field of the chosen models and can be handled well. We further reiterate here the scope of the UltraMNIST benchmark presented in this paper. Although we presented images of dimension 4000 × 4000, the motivation to design the benchmark comes from very large images. In this regard, it is certainly possible to generate a scaled version of UltraMNIST samples for even larger images (see https://github.com/transmuteAI/ultramnist). Moreover, it is possible to increase the complexity of the samples with an increased number of MNIST digits as well as more variations on the background, and this can be chosen based on the requirements of the problem. One important thing to note is that UltraMNIST is based on MNIST digits, which implies that the discriminative features of the models built on this dataset are not expected to be powerful enough to be used for transfer learning on natural images.

## Usage Notes

### Required packages

To run the baselines, a python version of 3.8 or higher is needed. In addition, it is recommended to use a pytorch version of 1.10 or higher. Full details related to installation and usage can be obtained from https://github.com/transmuteAI/ultramnist. For the pretrained models used in the baseline study, we use torchvision, and a version of 0.11 or higher is recommended.

### Terms of use, privacy, and license

This dataset is released under the terms of Creative Commons Attribution-Share Alike 3.0 license. It implies that any copies or modifications of this work should be released under the same or similar terms and not more restrictive terms.

### Can we use the full-resolution images directly?

Following the standard training scheme, we attempted to directly feed the full-scale images to our CNN model. However, we could only fit a batch size of 1 in the memory of a 32 GB NVIDIA V100 card with the EfficientNetB0 model. Hence, we conducted this experiment with the inclusion of gradient accumulation over 256 steps. Even with this, the convergence of the training process was difficult and extremely sensitive to the choice of the learning rate, and the best accuracy obtained on the test set was only 8.2%. Nevertheless, our conclusion from this experiment is that a 4000 × 4000 image is too big for the current models as well as the available GPU memory, and with the current possible setup, the downscaled images deliver better performance. With more effective training strategies, we hope to obtain better performance through processing the full-scale images directly.

### Can the randomly placed shapes cause spurious detections?

While adding the MNIST digits, we did not apply any interpolations at their edges when upscaling the digits. This leads to large-size staircases at the edges of these digits. However, the edges of the random shapes are smooth, with the size of the staircase being defined by the resolution of the image-grid. Thus, for almost all cases, the digits are different from the background shapes in at least one criterion. Zooming in on the UltraMNIST samples presented in Fig. 2 should provide a better understanding of this. Object-detection+classification-based methods ‘trained using the MNIST digits’ could achieve performance above 99.1% already on this dataset. A score of above 99% clearly indicates that the deep learning models can almost perfectly differentiate the MNIST digits from the spurious patterns and extract them from the large images.

### Is the 4000 × 4000 pixels size good enough to keep the benchmarking interesting in the long run?

With the current pace at which GPU resources are evolving, we certainly believe that it would soon be possible to fit those deep learning models into the memory that directly processes the full-size 4000 × 4000 image, and one might wonder if the presented benchmark would be relevant anymore or not. Clearly, this is not true. The intention of this paper is not limited to just presenting one dataset and letting researchers develop SOTA solutions on it. Rather, UltraMNIST is meant to be a benchmark for evaluating the performance of methods focused on handling very large images. To align with the fast developments related to GPU memory and its availability to a wide audience, we recommend researchers to conduct comparative experiments in the future on scaled-up variants of UltraMNIST as needed and to facilitate this, we have also open-sourced our data generation code.

### Is UltraMNIST a good proxy for actual applications?

We motivated the synthesis of the UltraMNIST dataset as well as the associated benchmark through real-world images from the field of nanoscopy. It is true that nanoscopic images might not be very close to our UltraMNIST images, however, we have given our best attempt to include the primary challenges of the nanoscopic dataset into UltraMNIST, while also limiting the complexity to an extent that it can be experimented by a broad audience. The primary challenges we are interested to tackle include handling very large images with CNN as well as ensuring that the semantic correspondence between objects of different scales as well as different spatial positions is preserved during processing. We have built UltraMNIST based on these motivations.

It can also be anticipated that the models built on UltraMNIST might not be directly transferable to other actual applications, and this could be due to the vast difference in properties of UltraMNIST compared to the actual ones. However, we also believe that novel methods built for handling UltraMNIST could already advance towards (although not be the solution) models needed for handling high-resolution images in actual applications. Further, the UltraMNIST benchmark is also going to be useful to study the properties of existing models that are designed for high-resolution image classification.

### Potential reuse of the data

We hope that the research work presented in this paper promotes further development in the following aspects.

#### Enhanced interpretation of very large images

With the presented benchmark dataset and the associated baselines, we hope to see the development of novel training strategies for CNNs that do not involve any downsampling of the input images. Solutions to such problems are of significant value to domains such as medical imaging and aerial imagery analysis, and we hope that apt solutions to the presented problem could add value in terms of extracting better semantic information as well as processing under limited computational memory resources when input images are very large.

#### Building extreme scale-agnostic models

Solutions to the UltaMNIST problem could answer the question of handling the extreme scale variations in any image. While CNNs learn invariance/equivariance to a certain extent, doing the same for scale variations beyond 1:1000 or more is currently not possible, and we hope the solutions to UltraMNIST could help achieve this.

#### Translating computational memory load into computational time load

In the current state, processing of very large images with CNNs leads to a bottleneck in terms of computational memory usage. Hardwares have a fixed amount of GPU memory, and CNN models processing very large images cannot fit into this memory space. We speculate that. the presented benchmark will lead to the development of new CNN training methods that scale the computational load in time dimension such that even very large problems can be handled with limited GPU memory.

## Implications of Noise

To determine the complexity of the noise within the UltraMNIST dataset, we conducted an experiment. We trained a Faster R-CNN model on noise-free training data and subsequently evaluated its predictive performance on a test dataset with incrementally added noise.

### FasterRCNN implementation

We utilized a pre-trained Faster R-CNN model with a ResNet-50-FPN backbone^31^. The model is trained with a learning rate of 1e-4 and an AdamW optimizer. The FasterRCNN model was trained in order to identify digits ranging from 0 to 9 across 10 classes in the UltraMNIST images. The model is trained for 50 epochs with batches of 16, maintaining the original image resolution of 4000 × 4000. We selected the model that demonstrated the lowest validation loss for inference. The digits with a score of ≥ 0.4 are considered as a predicted output. Finally, the correctly classified digits are those whose intersection over union (IoU) is at least 0.5 between the predicted and ground truth bounding boxes.

### Dataset Creation

We generated a noise-free dataset of 2800 images for both training and testing. The training dataset was divided into training and validation sets with an 80-20 split, respectively. For each image, an XML file containing labels and bounding box coordinates was created. The noise in the UltraMNIST dataset arises from the number of shape pairs (circles and triangles) and the checkerboard size. To assess the impact of shape pairs, we maintained a constant checkerboard size of 4 and varied the number of shape pairs (1 to 5) in the test datasets. Conversely, to evaluate the effect of checkerboard size, we fixed the shape pairs at 1 and varied the checkerboard sizes (4, 8, 12, 16, 20, and 24). The test datasets required a total of 12,254 predictions.

### Observations

**Fig. 6:**
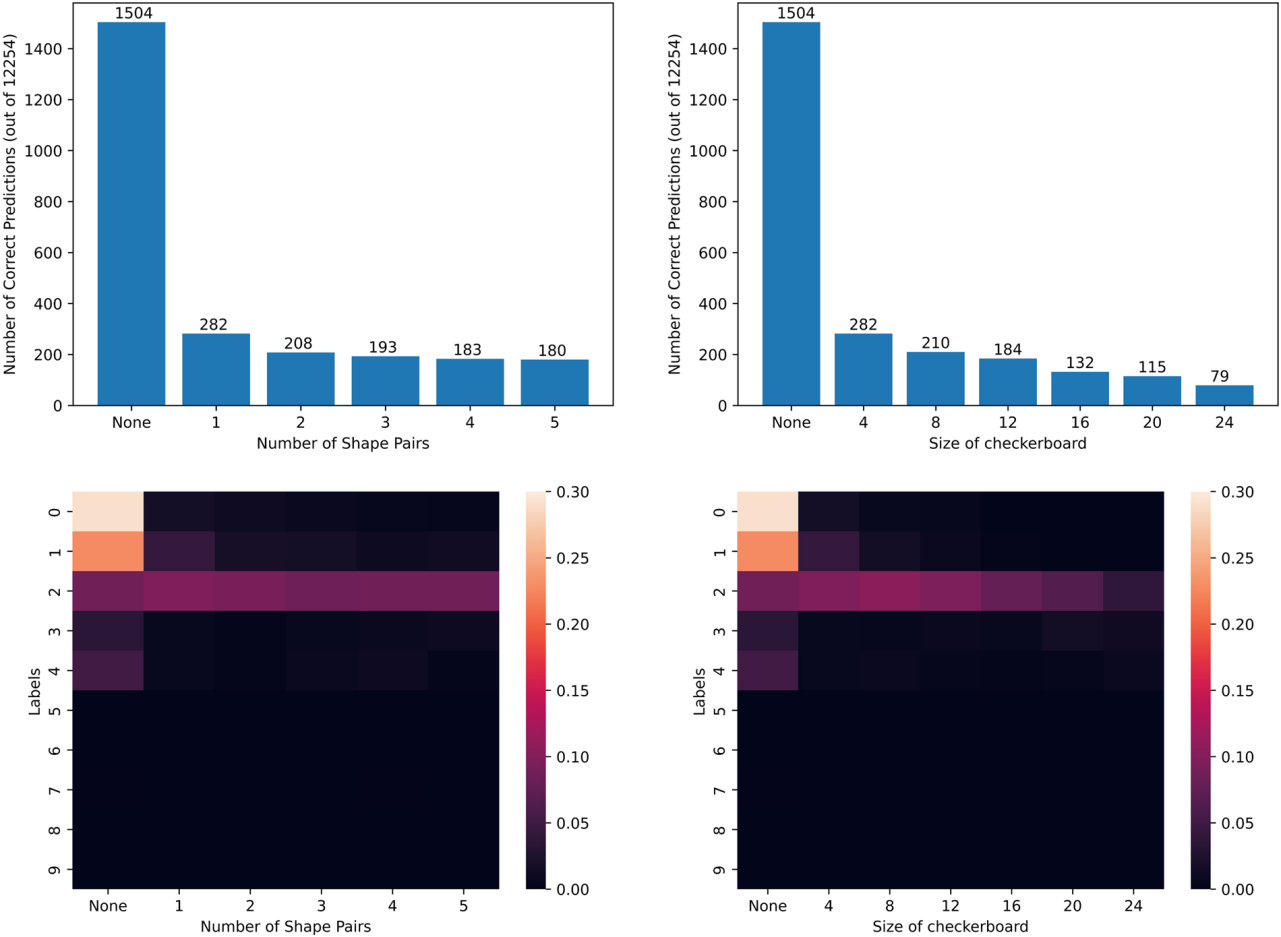
Implication of Noise: The values on top of the bar plot denote the number of correctly identified digits. The heatmap legend represents the proportion of correctly classified digits.

The Faster R-CNN model exhibits sub-optimal performance on the noise-free test dataset, accurately classifying merely 1504 out of 12254 digits. This may be attributed to the diverse sizes of the digits within the images. Additionally, the Faster R-CNN model predominantly classifies digits 0 and 1, sporadically classifies digits 2, 3, and 4, and fails to recognize digits 5 through 9, with only a single correct classification of the digit 7 (see Fig. 6). The implications of adding noise incrementally are:

- Increasing number of shape pairs: The number of correct predictions by the Faster R-CNN model decreases as the number of shape pairs increases, as seen in Fig. 6. The introduction of a single shape pair results in a drastic decrease in accuracy from 12.3% to 2.3%. Subsequent increases in the number of shape pairs result in progressively smaller reductions in accuracy. The lower-left image in Fig. 6 shows the relationship between the proportion of correctly classified digits and the number of shape pairs. The model’s accuracy in predicting the predominantly classified digits 0 and 1 significantly diminishes with an increasing number of shape pairs, a pattern that also extends to digits 3 and 4. However, interestingly, the model’s ability to classify the digit 2 does not deteriorate as significantly with an increase in the number of shape pairs.
- Increasing the size of the checkerboard: A pattern similar to that observed with the shape pairs is seen here, with the model’s performance deteriorating more rapidly in this scenario.

## Data Availability

**Data maintenance**. UltraMNIST^21^ can be downloaded from the official page hosted on the Kaggle platform and accessible at https://dataverse.no/dataset.xhtml?persistentId=doi:10.18710/4F4KJS. Information related to alternative download links will also be made available on our official page at https://github.com/transmuteAI/ultramnist. This page will also provide any future updates related to the dataset. **Benchmark and code**. Code related to all benchmark experiments can be obtained from our repository hosted on GitHub at https://github.com/transmuteAI/ultramnist.
